# When Does the Leader’s Positive Expression Get a Positive Response From Members? The Three-Way Interaction Effects of Perceived Deep/Surface Acting, Positive Affect, and Quality of Leader-Member Exchange on Work Engagement

**DOI:** 10.3389/fpsyg.2021.655047

**Published:** 2021-08-26

**Authors:** Sung Hyoun Hong, Min Soo Kim

**Affiliations:** School of Business, Hanyang University, Seoul, South Korea

**Keywords:** perceived deep acting, perceived surface acting, positive affect, quality of leader-member exchange, work engagement

## Abstract

Although researchers have argued that a leader’s positive affective display effectively induces work motivation among members, it has not always resulted in desirable outcomes. This research addresses these critical issues and explains why individuals react differently, by considering the three-way interaction of the characteristics of expression, the positive affect of the members, and quality of leader-member exchange (LMX). To verify our hypotheses, 698days from 47 leaders and 146 members were collected through the Experience Sampling Method. The analysis was conducted using HLM, and the results showed that, for members with high quality LMX, the positive effect of perceived deep acting on work engagement was strengthened when positive affect was high, and the negative effect of perceived surface acting was weakened when positive affect was high. On the other hand, members with low-quality LMX showed a stronger positive effect of perceived deep acting on work engagement when positive affect was high, and the negative effect of perceived deep acting was mitigated when positive affect was low. These results demonstrate that quality of LMX serves as a context of the affective display between leaders and members, and the effect of displaying positive affect relies on members’ perception of the characteristics of the expression and the affective state.

## Introduction

Many organizations and experts have highlighted that a leader’s affective display is an integral component of the leadership process (Ashkanasy et al., 2017). Affective display refers to all forms of affective expression expressed by an actor and includes verbal, non-verbal, and facial expressions (Van Kleef, 2016). In terms of emotional management, it is expected that positive affective display by leaders, in particular, will induce members’ desirable work-related outcomes, like motivation and engagement (George, 2000; Lin et al., 2016). The most traditional and predominant explanation for this is the contagion effect of affect, according to which, through affective display, the positive affect by a leader will be transmitted to members, thereby increasing the latter’s work motivation (Hatfield et al., 1994; Bono and Ilies, 2006). Empirically, some studies have demonstrated that positive affective display increases psychological safety, job resources, and proactive behaviors of members (Liu et al., 2017; Cooper et al., 2018).

While significant efforts have been made in recent years to understand the role and the mechanism of a leader’s positive affective display, many researchers have underlined potential problems and argued that the effects on members cannot be explained solely by the contagion effect. Specifically, several studies have shown that a leader’s display of positive affect can decrease members’ efforts at work and result in negative work behavior depending on the situation (Gaddis et al., 2004; Sy et al., 2005; Visser et al., 2013). Therefore, this study deals with the research question of why a leader’s positive expressions may not always engender desirable results for subordinates.

To address this paradox, we draw upon the emotion as social information (EASI) theory (Van Kleef, 2009, 2016), which states that social information is contained in displayed affect, so that observers form responses based on the judgment of this information. Specifically, this theory emphasizes that the observer’s reaction can be altered depending on the characteristics of expression, context, and self. Accordingly, members’ work-related response is induced not only by the valence of the displayed affect, but by their own understanding of the expression (Groth et al., 2009; Wang and Seibert, 2015). In addition to the authenticity of the affective display, since interaction with a leader is a daily occurrence in a lasting relationship, members make a judgment based on the established relationship with leaders (Fisk and Friesen, 2012; Liu et al., 2017). Moreover, as the affective state serves as a background of reaction to workplace events, a member’s work-related response to a leader’s positive affective display can alter in accordance with the affective state of the member (Forgas, 1995; Janssen et al., 2010).

The purpose of this research is to divide a leader’s positive affective display into whether it is perceived as deep acting or surface acting, and to examine three-way interaction effects by quality of LMX (leader-member exchange) and positive affect for each. The nature of the relationship serves as the fundamental background of interaction, influencing the meaning and social impact of the affective display (Van Kleef et al., 2010b). Therefore, we consider quality of LMX as a context, suggesting that the quality of LMX encourages members to interpret the meaning of deep and surface acting differently. In addition, we argue that a member’s work response is also dependent on the positive affect.

Additionally, we consider the level of analysis in order to verify the effect of a leader’s positive affective display on members elaborately. Firstly, a leader’s affective display and members’ positive affect are essentially a within-person-level phenomenon. Affective display is inconsistent because it is the reactive aspect of a leader’s workplace events (Cropanzano et al., 2017). The affective state of the members also fluctuates every moment as the circumstances around them change (Beal and Ghandour, 2011). Since LMX is stable and established differently across the members, it should be considered at the between-person level (Henderson et al., 2009). Consideration of the within-person level reflects the complex changing situations, enabling accurate capture of the interpersonal effects of the affective display (Diefendorff et al., 2019). Accordingly, we address the affective display and members’ reactions at the within-person level and consider quality of LMX as a between-person-level moderator and positive affect as a within-person-level moderator.

The implications of our research are as follows. The current paper contributes to the leader’s affective display literature by demonstrating how a leader’s positive affective display influences members comprehensively. Specifically, we highlight how members’ response to displayed affect can differ depending on the perception of expression, positive affect, and quality of LMX. Second, this study links the leadership field with the work engagement field by demonstrating the role of a leader in members’ work engagement experience through a multilevel perspective. Finally, we indicate that in order to understand the interpersonal effect of affective display, it is imperative to consider the observer’s affective state and that in the continuing relationship between the leader and the member, quality of LMX serves as an important relational context. These findings contribute toward expanding the field of affective display and the EASI theory.

## Theoretical Background and Hypotheses

### Members’ Reaction to Leader’s Affective Display

According to the EASI theory (Van Kleef, 2016), members capture the social information from the affect displayed and determine their work behavior based on that information. Social information comprises a leader’s intention, orientation, or judgment of member’s work (Visser et al., 2013; Wang et al., 2018). For example, depending on the situation, a member will likely believe that the leader has displayed a positive affect to provide encouragement or convey sarcasm; the various expressions could induce diverse work responses depending on the interpretation. It should be borne in mind that the valence of affect does not regulate this cognitive process; rather, it is based on the characteristics of expression, relational context, and the state of the members, which act as the cue to make a judgment (Van Kleef et al., 2010b).

In this study, we distinguish the type of positive affective display in terms of deep and surface acting, depending on the expression’s authenticity. These concepts are typically used in the emotional labor field. Deep acting refers to a leader displaying a more natural and genuine affect, while surface acting means the leader is concealing the true affect and expressing a fake affect (Humphrey et al., 2008; Humphrey, 2012; Grandey et al., 2013). Therefore, if a member perceives the leader’s positive affective display as deep acting, it will be considered affective support for the members and used as a social resource to build motivation and accomplish tasks (Gardner et al., 2009; Fisk and Friesen, 2012). However, if it is perceived as surface acting, the member will believe that the leader has deceived him/her and evaluate the leader’s hidden intention in a negative way (Groth et al., 2009). Consequently, the member’s work morale may decrease and he/she may be reluctant to behave for the leader (Hu and Shi, 2015; Hideg and Van Kleef, 2017).

In conjunction with the type of expression, members’ cognitive judgments depend on the nature of their relationship with the leader (Van Kleef et al., 2010a). Unlike other settings (e.g., customer service), affective expression among internal members (especially the leader) has a more complex – but powerful – impact, because they have a rather long-lasting relationship (Gooty et al., 2010; Ashkanasy et al., 2017). LMX refers to the quality of the exchange relationship established between the leader and individual members. The quality of LMX varies across members (Graen and Uhl-Bien, 1995). The LMX theory suggests that, in contrast to low-quality LMX characterized by economic exchange, high-quality LMX features trust, support, and loyalty, whereby members express emotional attachment toward their leader (Liden et al., 1997; Henderson et al., 2009). In addition, members with high-quality LMX are more likely to assess the leader’s expression favorably; they will also likely regard emotional support as more than a mere role of a leader and reciprocate accordingly (Martin et al., 2018). Therefore, the quality of LMX, as a relational context, can make a difference in the meaning and interpretation of the expression that members accept.

Finally, a member’s affective state should also be considered in order to understand his/her reaction. Positive affect refers to the extent to which an individual feels enthusiastic, excited, and interested, and fluctuates every moment (Watson et al., 1988). When positive affect is high, individuals are full of energy, confident in their work, and tend to view the target with optimism (Lopez et al., 2018). According to Forgas’ (1995), affect serves as the basis for forming individual responses. In a positive state, members heuristically judge the leader’s expression more positively and respond accordingly. Meanwhile, some scholars argue that positive affect functions as a personal resource, enriching job resources and making better use of available resources (Janssen et al., 2010; McGrath et al., 2017). The broaden-and-build (B&B; Fredrickson, 2001) theory elucidates that positive affect extends the scope of an individual’s interest, thinking, and behavior to bring in resources and manage them on his/her own in order to cultivate motivation for work. For example, optimistic thinking increases resilience, while social support gained through interactions is used as a resource for work (Gorgievski et al., 2011; Lopez et al., 2018). Based on the arguments above, we propose that positive affect represents a member’s state at the moment when the leader displays positive affect; concurrently, it alters the member’s judgment of the expression.

### Leader’s Positive Affective Display and Work Engagement

In this study, we focus on work engagement as a member’s desirable outcome of a leader’s positive affective display. Work engagement is defined as a positive, fulfilling state of mind for one’s work, and affectively highly a motivated state (Schaufeli and Bakker, 2004). The importance of work engagement has been emphasized because engaged members behave proactively to enhance the organization’s effectiveness and contribute to productivity (Xanthopoulou et al., 2009; Rich et al., 2010; McGrath et al., 2017). Previous studies have characterized a leader as an emotional manager, in an effort to increase members’ engagement (Christian et al., 2011; Tanskanen et al., 2019). It is expected that positive expressions from the leader will induce members’ positive affect and foster ambition at work (Goswami et al., 2016; Cooper et al., 2018). Therefore, we aim to reveal when and how a leader’s positive affective display can trigger members’ work engagement. We divide the context into high- and low-quality LMX, and discussed how perceived deep and surface acting of a positive expression impact work engagement according to a member’s positive affect.

We also argue that members with high-quality LMX regard a leader’s positive affective display as a more valuable form of social support, which has been perceived as deep acting. This is attributable to affective support from the leader, who trusts and supports such members (Liden et al., 1997). Members will evaluate this as more than just social support and consider the interaction with a leader to bring in more valuable job resources, which they can use for their work (Dasborough and Ashkanasy, 2002; Martin et al., 2018). Accordingly, they can be driven to become absorbed in their work in return for support, as they feel more responsible for the leader than members with low-quality LMX (Henderson et al., 2009). Furthermore, regarding this influence, we predict that this positive affect will be strengthened when members are in a positive affective state. According to positive psychology research, members with a positive affective state can better utilize the resources gained to achieve their own work goals (Janssen et al., 2010; Knight et al., 2017). In a positive state, for example, members can expand their resources beyond themselves and use them for work, unlike a relatively not positive state when members use acquired social resources to improve their current affective state (Fredrickson, 2001). Therefore, in a positive affective state, members can manage resources more successfully and can become engaged in their work based on abundant job resources in order to return the leader’s support (Lyubomirsky et al., 2005; Eisenberger et al., 2014). Drawing on this logic, we derive the following hypothesis:

H1: *For members with high-quality LMX, the positive effect of perceived deep acting on work engagement will be strengthened when the positive affect is high compared to when it is low*.

Meanwhile, a positive affective display perceived as surface acting makes members believe that the leader is not genuinely positive and has conveyed a fake affect (Gardner et al., 2009). Although the leader has been deceitful, members with high-quality LMX are more likely to judge the leader’s intention favorably, and will conclude that there is a problem with the current state based on information about the actual affect (Fisk and Friesen, 2012; Van Kleef, 2016). Simultaneously, we assert that if members experience this interaction in a non-positive state, work engagement will diminish, but in a positive state, the negative influence of the perceived surface acting on work engagement will be mitigated. Since members who are not in a positive state lack available resources, it is difficult to become engaged in work without external assistance (Hobfoll, 1989). Specifically, based on perceived surface acting, it is challenging to experience work engagement, since members believe that they cannot receive resources from the leader, and that there is a problem with the work situation (Halbesleben et al., 2014; Uy et al., 2017). However, in a positive affective state, members assess the potential work situation and leader’s intention more optimistically, thereby alleviating the negative effects of inference (Ortony et al., 1990; Lopez et al., 2018). Furthermore, in such a state, members devote abundant resources to perform their work roles and strive to solve the presumed problems for the leader (McGrath et al., 2017). Consequently, the inference of the surface acting is less likely to hinder motivation, and the negative effect of perceived surface acting on work engagement will decline. Accordingly, we suggest the following hypothesis:

H2: *For members with high-quality LMX, the negative effect of perceived surface acting on work engagement will be weakened when the positive affect is high compared to when it is low*.

In contrast, members with low-quality LMX will view the perceived deep acting of positive expression as a leader’s role merely. These members only form transactional relationships, so they do not see the cause of genuine expression as favorable, like members with high-quality LMX, because they believe the leader will not act beyond his/her obligations toward them (Dasborough and Ashkanasy, 2002; Eberly and Fong, 2013). Therefore, perceived deep acting will affect a member’s work engagement as no more than a single social resource. When positive affect which promotes the inflow of resources is high, members retain abundant resources without the leader’s support (Fredrickson, 2001). Since positive affect increases job resources (psychological resources, like resilience, and social resources, such as social support) to facilitate engagement, members develop a high level of motivation on their own (Gorgievski et al., 2011; McGrath et al., 2017). In addition, as positive affect helps members to continue their work behavior, the positive effect of perceived deep acting on work engagement will be reduced (Carver and Scheier, 1990). Meanwhile, in a low positive affective state, where resources are scarce and it is challenging to remain motivated, a leader’s support is the only means of conserving job resources (Janssen et al., 2010; Uy et al., 2017). Accordingly, since members actively attempt to use a leader’s resources to experience work engagement (Halbesleben et al., 2014), perceived deep acting will positively influence work engagement more than when positive affect is high. These arguments lead to the following hypothesis:

H3: *For members with low-quality LMX, the positive effect of perceived deep acting on work engagement will be weakened when the positive affect is high compared to when it is low*.

We argue that a leader’s displayed positive affect perceived as surface acting to members with low-quality LMX may impel them to make negative inferences and express pessimistic work reactions. They are more likely to perceive a leader’s deceitful behavior as inappropriate and their intentions as negative (Hideg and Van Kleef, 2017). Consequently, these members will be dissatisfied with the interaction and demonstrate a negative attitude toward work, in addition to low morale (Hu and Shi, 2015). However, when members’ positive affect is low, the negative effect of perceived deep acting on work engagement will not be significant because the members have not already been engaged in their work. According to Hobfoll (1989), when resources are inadequate, individuals focus more internally on preserving their own resources. Hence, it is already impracticable for members to experience work engagement since they do not have adequate resources for their work, and there is less room for perceived surface acting to hamper the experience of engagement (Halbesleben et al., 2014; Zacher et al., 2019). Meanwhile, in a positive state, the negative effect of perceived surface acting will be revealed, and members’ engagement will be lowered through a negative judgment (Fisk and Friesen, 2012). Members are in a state of cultivating work motivation through a highly positive affect, but are hindered by the leader’s positive affect expressed in surface acting, making it harder to continuously remain absorbed in work. Therefore, we posit the following:

H4: *For members with low-quality LMX, the negative effect of perceived surface acting on work engagement will be weakened when the positive affect is low compared to when it is high*.

**Figure 1:**
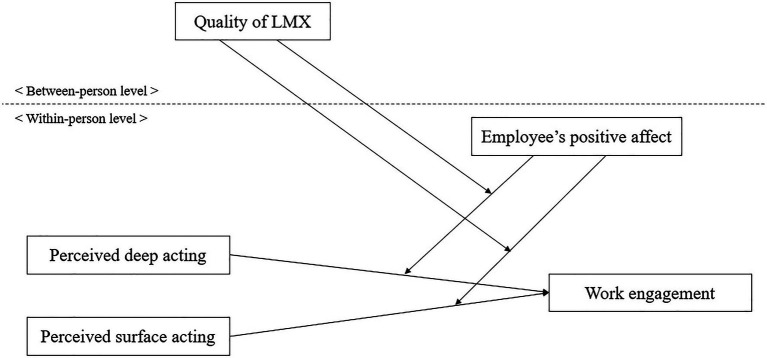
Proposed research model.

Figure 1 displays our research model.

## Materials and Methods

### Procedure and Sample

We collected multilevel data through the Experience Sampling Method (ESM) to examine the hypotheses of this study. The ESM is a data collection method that measures participants’ responses repeatedly on a daily or weekly basis (Larson and Csikszentmihalyi, 1983; Fisher and To, 2012), and is a reasonable way to accomplish the purpose of our study, especially since it has been recognized as the most valid way to measure interactions, affects, and daily work experiences occurring in the workplace (Dimotakis et al., 2011; Podsakoff et al., 2019). Moreover, this approach reduces bias and errors that arise from respondents’ recalling memories, so that research studies have the advantage of more accurately capturing respondents’ experiences and within-person phenomena (Schwartz et al., 2009).

However, the ESM could result in selective nonresponse for participants (Fisher and To, 2012). To compensate for these potential problems, we conducted a survey of the teams that could participate on a daily basis and informed the manager about it in advance. Financial incentives were offered to increase the motivation of participants to respond, and we met them to explain about the survey. We attached an explanatory sentence in the questionnaire so that the participants could recall and clearly understand the survey; moreover, we also sent a reminder on a daily basis during the survey period, to encourage participation. In addition, the responses were completed at the end of work time around 4 or 5pm, and the participants recorded the time of completion of the questionnaire. We also announced that the confidentiality of the participants will be maintained.

This study collected data from 36 organizations pertaining to various industries, including manufacturing, finance, and service, in South Korea. To enhance quality, teams consisting of one leader and three or more members were selected. We met with the participants in advance and distributed the questionnaires directly, and visited them again on the last day of the survey. The survey was conducted with pencil-and-paper questionnaires, consisting of different sections depending on the characteristics of the variables. For example, variables, such as LMX, were measured before the daily variables were measured, and after which participants responded to questions about daily affective display and state. Finally, we collected data for 1week (five consecutive workdays). Moreover, we categorized the questionnaires into types – for leaders and members, and according to the level of the variables – in order to reduce method biases (Podsakoff et al., 2003). Overall, we recruited 47 leaders and 146 members from 47 teams as participants and collected a total of 698days as final data points.

Regarding the characteristics of our sample related to leaders, 59.57% were male; the average age was 46.37 (SD=8.32); 51.06% graduated from college; the average team size was 7.51 (SD=5.14); and the industry types were 25.53% in service, 19.15% in manufacturing, 10.64% in finance, 2.10% in distribution, and 42.58% from other industries. With regard to the members, 54.42% were male; the average age was 35.27 (SD=7.95); 73.3% had received college education; and the average period that they had worked with the leader was 3.19 (SD=4.62) years.

### Measure

The continuous variables were measured using a 1–5-point Likert scale (1=strongly disagree; 5=strongly agree).

### Within-Person Level

#### Work Engagement

This study measured the work engagement of members through five items developed by Schaufeli et al. (2002), and members responded to their engagement every day. The example items are as follows: “Today, I am enthusiastic about my work,” “Today, I am immersed in my work,” and “Today, at my work I always persevere, even when things do not go well” (Cronbach’s alpha=0.85).

#### Perceived Deep Acting

We measured perceived deep acting by utilizing three items developed by Diefendorff et al. (2005) for daily measurement. Members responded, and a sample item was: “Today, when a leader displayed positive affect toward me, the leader tried to actually experience the displayed affect” (Cronbach’s alpha=0.89).

#### Perceived Surface Acting

Perceived surface acting was also answered daily by the members, and four items were utilized, as suggested by Diefendorff et al. (2005). A sample item was: “Today, when a leader displayed positive affect toward me, the leader showed feelings that seemed different from what the leader actually felt” (Cronbach’s alpha=0.89).

#### Positive Affect

The members responded to their daily positive affective state, which is defined as the state of being alert, happy, and excited (Watson et al., 1988), and measured the level of specific positive affect. Four items from the PANAS scale (Watson et al., 1988) were used, and example items were “happy” and “excited” (Cronbach’s alpha=0.93).

#### Control Variables

We used the leader’s positive affective display and recovery as control variables at the within-person level. A leader’s positive affective display was measured utilizing the Positive and Negative Affect Schedule (PANAS; Watson et al., 1988) scale, based on the suggestion from Bono and Ilies (2006). Four items were used, and one of the examples of the items was “Today at work, the leader displayed happy to me” (Cronbach’s alpha=0.92). We also controlled recovery which is highly associated with work engagement to exclude alternative explanations. Recovery was measured using three items from Sonnentag (2003) and one time of sleep quality suggested by Buysse et al. (1989). Sample items are as follows: “Last night, I had enough time to recover from the day’s work” and “The quality of sleep was good today” (Cronbach’s alpha=0.93).

### Between-Person Level

#### Quality of LMX

This study used seven items developed by Liden and Maslyn (1998) and applied it to members. A sample question was as follows: “My leader is a lot of fun to work with” and “I do work for my leader that goes beyond what is specified in my job description” (Cronbach’s alpha=0.95).

#### Control Variables

To enhance the validity of this study, the following between-person level variables were controlled on the basis of prior research. We included members’ age, education, occupation type, position, personality (extraversion), gender dissimilarity with the leader, and dyadic tenure with the leader. In addition, the averages of the major within-person level variables were included in the analysis to control the effect on the work engagement. Specifically, the demographic characteristics were dummy-coded with 1 for the most significant portion and 0 for the others, and for education, 1 for bachelor’s degree; for occupation type, 1 for office clerk; for a position, 1 for staff; and for gender dissimilarity, 1 for different and 0 for the same. Extraversion was measured by five items from Goldberg’s (1999) International Personality Item Pool (IPIP) Big Five scale, and the example is “I make friends easily.”

### Team (Leader) Level

#### Control Variables

We included the leader’s age, industry type, team size, leader’s extraversion, and positive affect in analyzing our hypotheses, and measured them form the leaders. Industry type has dummy-coded the service industry with 1, and extraversion was measured using five items from the IPIP scale (Goldberg, 1999). The positive affect of the leader was measured daily for five consecutive days using four items of the PANAS scale (Watson et al., 1988).

### Analytic Strategy

We first conducted a multilevel confirmatory factor analysis (MCFA) to establish the discriminant validity of our variables using Mplus 7.0 (Muthén and Muthén, 2017). To verify the validity of the used within-personal-level variables, the proportion of within-person variance was checked as recommended by Podsakoff et al. (2019). Subsequently, since the data of this study included the multilevel structure, three-level random coefficient modeling was performed using HLM 6.02 (Raudenbush et al., 2004) to examine the hypotheses. To enhance the validity of the analysis, we performed group-mean centering for all the used within-personal level variables, and the quality of LMX was grand-mean centered to reduce the potential collinearity problem (Hofmann and Gavin, 1998; Raudenbush and Bryk, 2002). In addition, implementing group-mean centering can reduce potential endogeneity concerns that may occur due to omitted variables (Antonakis et al., 2019). Furthermore, as we intended to verify the three-way interaction effect, the analysis was performed by hierarchical procedures that include control variables, predictors, interaction terms, and three-way interaction terms in turns.

## Results

### Descriptive Statistics and Multilevel Confirmatory Factor Analysis

Table 1 shows the means, standard deviations, and correlations of the variables used in this study. A series of MCFAs was conducted on five within-person-level variables and one between-person-level variable (leader’s positive affective display, work engagement, perceived deep acting, perceived surface acting, positive affect, and quality of LMX). Our model [five factors at within-level, six between-level; *χ*^2^(469)=991.02, root mean square error of approximation (RMSEA)=0.03, comparative fit index (CFI)=0.94, Turker-Lewis index (TLI)=0.93] indicates a better fit to our data than alternative models; model A [four factors at within-level, five between-level; combined perceived deep acting and perceived surface acting; *χ*^2^(478)=1766.19, RMSEA=0.06, CFI=0.86, TLI=0.84]; model B [three factors at within-level, four between-level; additionally combined work engagement and positive affect; *χ*^2^(485)=2222.23, RMSEA=0.07, CFI=0.80, TLI=0.78]; model C [two factors at within-level, three factors at between-level; additionally combined leader’s positive affective display with perceived deep acting and perceived surface acting; *χ*^2^(490)=3281.11, RMSEA=0.09, CFI=0.69, TLI=0.65]; model D [one factor at within-level, two between-level; combined all within-person level variables; *χ*^2^(493)=4920.00, RMSEA=0.11, CFI=0.50, TLI=0.45]; and model E [one factor at within-level, one factor at between-level; combined all variables; *χ*^2^(494)=5078.40, RMSEA=0.11, CFI=0.48, TLI=0.43]. Regarding the goodness-of-fit index, Hu and Bentler (1999) recommended that the model is acceptable when RMSEA<0.05, CFI>0.90, and TLI>0.90. Consequently, since our model passed this cutoff criterion, the discriminant validity for our variables has been confirmed.

**Table 1:** Mean, standard deviation, and correlations.

Team-level variables	Mean	SD	1	2	3	4							
1. Leader’s age	46.37	8.32											
2. Industry type^a^	0.26	0.44	0.04										
3. Team size	7.51	5.14	0.03	0.04									
4. Leader’s extraversion	3.46	0.58	0.12	0.10	0.14								
5. Leader’s positive affect	3.35	0.60	0.30^*^	0.26	0.11	0.49^**^							
**Between-person variables**	**Mean**	**SD**	**1**	**2**	**3**	**4**	**5**	**6**	**7**	**8**	**9**	**10**	**11**
1. Age	35.27												
2. Gender dissimilarity^b^	0.39	0.49	0.07										
3. Education^c^	0.73	0.44	−0.16	−0.06									
4. Occupation type^d^	0.65	0.48	0.11	0.20^*^	0.11								
5. Position^e^	0.64	0.48	−0.21^**^	−0.10	−0.13	−0.11							
6. Tenure (with leader)^f^	3.19	4.62	0.47^**^	−0.10	−0.19^*^	−0.02	−0.30^**^						
7. Extraversion	3.34	0.83	0.01	0.14	−0.06	−0.03	−0.01	0.03					
8. Quality of LMX	3.56	0.91	−0.01	−0.13	−0.13	−0.00	0.12	−0.00	0.08				
9. PA display (mean)	3.58	0.60	0.06	−0.06	−0.07	0.05	0.18^*^	−0.07	0.14	0.68^**^			
10. Perceived deep acting (mean)	3.45	0.63	0.01	−0.12	−0.09	0.07	0.16	0.08	0.12	0.68^**^	0.59^**^		
11. Perceived surface acting(mean)	2.41	0.62	0.03	0.14	0.10	0.01	−0.10	−0.07	0.12	−0.61^**^	−0.49^**^	−0.68^**^	
12. Positive affect (mean)	3.19	0.64	0.06	0.06	−0.12	−0.07	0.02	0.01	0.36^**^	0.44^**^	0.63^**^	0.33^**^	−0.19^*^
**Within-person variables**	**Mean**	**SD**	**1**	**2**	**3**	**4**	**5**						
1. Recovery	3.19	0.86											
2. PA display	3.60	0.77	0.32^**^										
3. Work engagement	3.36	0.63	0.46^**^	0.41^**^									
4. Perceived deep acting	3.47	0.76	0.22^**^	0.45^**^	0.42^**^								
5. Perceived surface acting	2.40	0.75	−0.14^**^	−0.30^**^	−0.30^**^	−0.54^**^							
6. Positive affect	3.25	0.76	0.51^**^	0.47^**^	0.58^**^	0.26^**^	−0.16^**^						

*N* =698 (within-person), 146 (between-person), and 47 (team).

a Service industry=1; others=0.

b Different=1; same=0.

c Bachelor’s degree=1; others=0.

d Office clerk=1; others=0.

e Staff=1; others=0.

f Scale is year.

* *p* < 0.05;

** *p* < 0.01.

### Partitioning of Variance

Before testing our hypotheses, we conducted null model analyses along with MCFA to confirm the validity of the within-person-level variables. The null model analysis divides the variances of the variable into the within-person, between-person, and team levels, showing whether reasonable variances are distributed at each level, thus justifying the use of multilevel analysis, such as HLM. Accordingly, we have examined whether there are significant within-person variances in the four main and one critical within-person-level variables. Based on the results of Table 2, all variables displayed significant variances in the within-person level and the proportion of within-person variance ranged from 38.98 to 47.54% (leader’s positive affective display=47.54%; work engagement=39.53%; perceived deep acting=41.67%; perceived surface acting=38.98%; and positive affect=40.98). In addition, all the variables contained significant variances in the between-person (the proportion ranged from 24.59 to 44.07%) and team (leader) levels (the proportion ranged from 16.95 to 28.33%). Therefore, we have confirmed that our variables can represent within-person-level phenomena and that HLM is a valid analytical technique for our research.

**Table 2:** Variance components of null models for within-person-level variables.

Variables	Within-person-level variance (e^2^)/percentage	Between-person-level variance (r^2^)/percentage	Team-level variance (u^2^)/percentage
PA display	0.29^**^/47.54%	0.15^**^/24.59%	0.17^**^/27.87%
Work engagement	0.17^**^/39.53%	0.16^**^/37.21%	0.10^**^/23.26%
Perceived deep acting	0.25^**^/41.67%	0.18^**^/30.00%	0.17^**^/28.33%
Perceived surface acting	0.23^**^/38.98%	0.26^**^/44.07%	0.10^*^/16.95%
Positive affect	0.25^**^/40.98%	0.20^**^/32.79%	0.16^**^/26.23%

*The percentage of variance at within-person level in the variable was calculated as e^2^/(e^2^+r^2^+u^2^); the percentage of variance at between-person level in the variable was calculated as r^2^/(e^2^+r^2^+u^2^); and the percentage of variance at the team level in the variable was calculated as e^2^/(e^2^ + r^2^+u^2^)*.

* *p*<0.05;

** *p*<0.01.

### Hypothesis Testing

**Figure 2:**
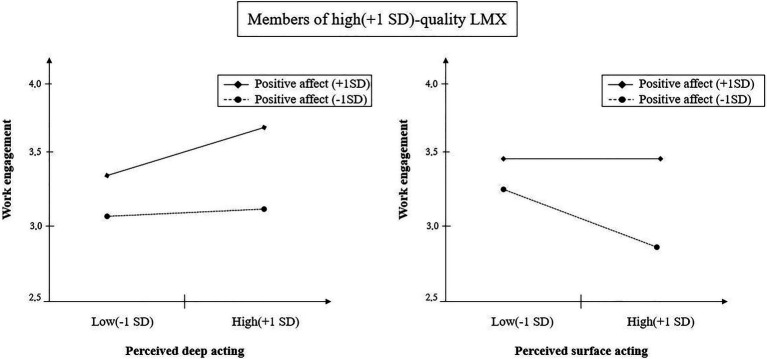
Three-way interaction slopes with work engagement as the dependent variable for members with high-quality LMX.

The results of HLM analysis are shown in Table 3. Hypothesis 1 predicted that for members of high-quality LMX, the positive effect of perceived deep acting on work engagement will be strengthened by positive affect. Based on Model 4 of Table 3, a three-way interaction term among perceived deep acting, positive affect, and quality of LMX was shown to be significant (*b*=0.47, *p*<0.05). We examined a two-dimensional graph to check the specific aspects, and the perceived deep acting was more strongly positively associated with work engagement when the positive affect was high, compared to when it was low (Figure 2). As a result, hypothesis 1 was supported.

**Table 3:** Results of HLM regressions: dependent variables are work Engagement.^a^

Variables^b^	Model 1	Model 2	Model 3	Model 4
**Team level variables**				
Intercept	0.61(0.66)	0.41(0.62)	0.46(0.64)	0.23(0.65)
Leader’s age	0.01(0.01)	0.01(0.01)	0.01(0.01)	0.01(0.01)
Industry type	0.12(0.10)	0.13(0.10)	0.12(0.09)	0.13(0.09)
Team size	0.02(0.01)	0.03(0.01)	0.03(0.01)^*^	0.03(0.01)^*^
Leader’s extraversion	0.08(0.14)	0.16(0.14)	0.16(0.14)	0.19(0.14)
Leader’s positive affect	−0.17(0.12)	−0.28(0.12)^*^	−0.27(0.12)^*^	−0.30(0.12)^*^
**Between-person variables**				
Age	0.01(0.01)^*^	0.02(0.01)^**^	0.02(0.01)^**^	0.02(0.01)^**^
Gender dissimilarity	0.14(0.01)	0.15(0.09)	0.12(0.09)	0.12(0.09)
Education	−0.06(0.10)	−0.03(0.09)	−0.06(0.09)	0.00(0.10)
Occupation type	−0.22(0.11)^*^	−0.23(0.11)^*^	−0.26(0.11)^*^	−0.30(0.11)^*^
Position	0.04(0.09)	0.07(0.08)	0.00(0.08)	0.03(0.09)
Tenure (with leader)	−0.04(0.02)^*^	−0.03(0.01)^*^	−0.04(0.02)^**^	−0.04(0.02)^*^
Extraversion	0.09(0.06)	0.05(0.06)	0.04(0.06)	0.02(0.06)
PA display (mean)	−0.11(0.13)	−0.19(0.13)	−0.13(0.12)	−0.11(0.12)
Perceived deep acting (mean)	0.33(0.09)^**^	0.44(0.11)^**^	0.38(0.11)^**^	0.35(0.11)^**^
Perceived surface acting (mean)	−0.03(0.08)	−0.09(0.08)	−0.09(0.08)	−0.08(0.08)
Positive affect (mean)	0.34(0.13)^*^	0.36(0.11)^**^	0.36(0.12)^**^	0.40(0.12)^**^
Quality of LMX		0.02(0.09)	0.04(0.09)	0.07(0.09)
**Within-person variables**				
Recovery	0.15(0.05)^**^	−0.02(0.05)	−0.00(0.04)	−0.02(0.04)
PA display	0.01(0.05)	0.03(0.05)	0.08(0.05)	0.08(0.05)
Perceived deep acting		0.18(0.06)^**^	0.16(0.06)^**^	0.18(0.06)^**^
Perceived surface acting		−0.10(0.08)	−0.02(0.07)	0.01(0.07)
Positive affect		0.31(0.06)^**^	0.32(0.06)^**^	0.31(0.06)^**^
**Two-way interaction**				
PDA×PA			−0.35(0.12)^**^	−0.44(0.14)^**^
PDA×Quality of LMX			−0.09(0.05)	−0.12(0.06)
PSA×PA			−0.32(0.12)^**^	−0.20(0.15)
PSA×Quality of LMX			−0.07(0.08)	−0.09(0.08)
PA×Quality of LMX			0.02(0.05)	0.06(0.05)
**Three-way interaction**				
PDA×PA×Quality of LMX				0.47(0.18)^*^
PSA×PA×Quality of LMX				0.35(0.11)^**^

a *p<0.05; ^**^p<0.01. Numbers outside parentheses are the coefficient, and numbers in parentheses are the standard error.

b PDA refers to “Perceived deep acting,” PSA refers to “Perceived surface acting,” PS refers to “Positive affect,” and LMX refers to “Leader-member exchange.”

Hypothesis 2 was about the three-way interaction effect among perceived surface acting, positive affect, and quality of LMX, and for members of high-quality LMX, the negative effect of perceived surface acting on work engagement would be mitigated when positive affect is high. In line with our prediction, the three-way interaction effect was significant (model 4 of Table 3; *b*=0.35, *p*<0.01), and the interaction figure is shown in Figure 2. The negative effect of perceived surface acting appeared when positive affect was low, but it was found to decrease when positive affect was high. Therefore, hypothesis 2 was also supported.

**Figure 3:**
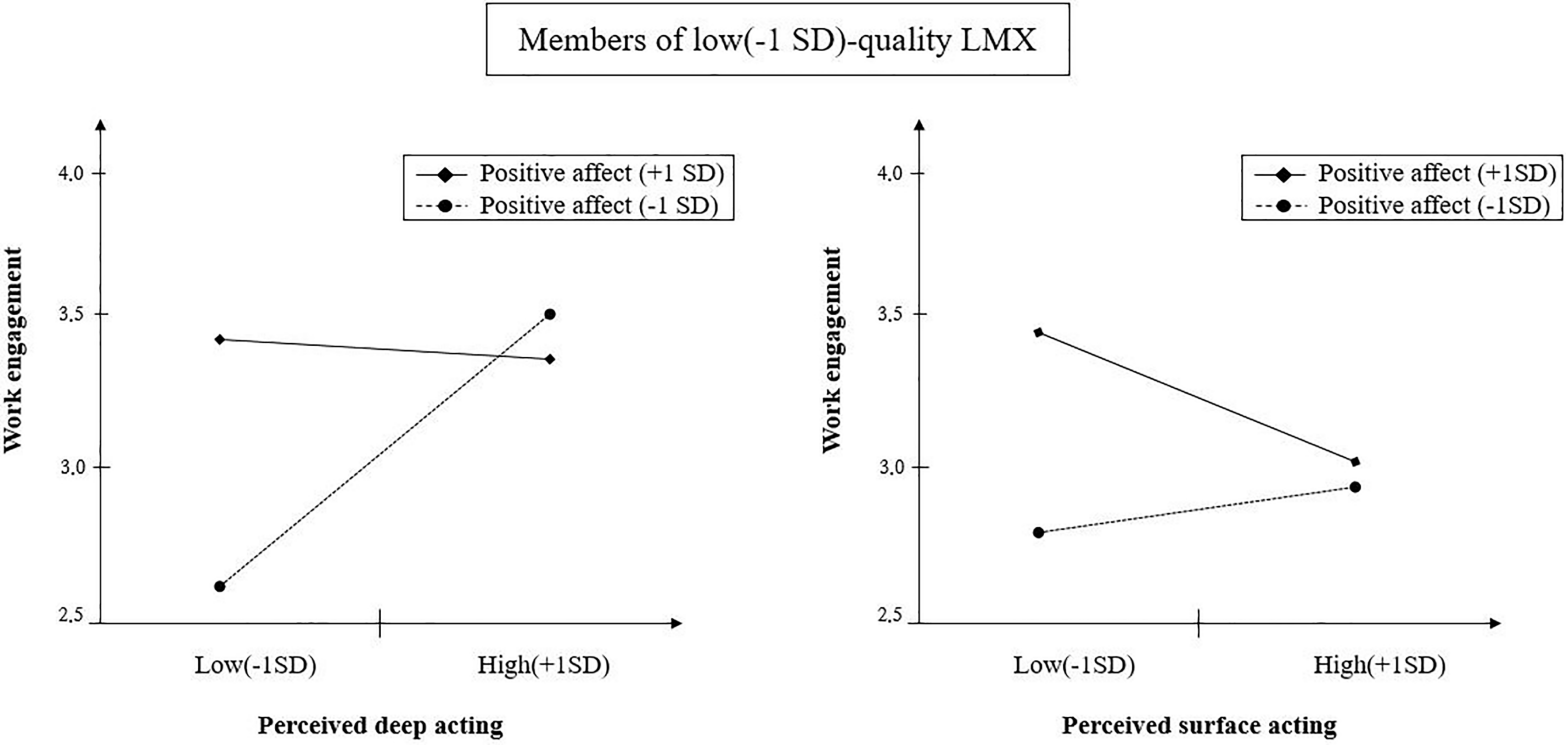
Three-way interaction slopes with work engagement as the dependent variable for members with low-quality LMX.

Hypotheses 3 and 4 are related to members of low-quality LMX, and hypothesis 3 suggested that the positive effect of perceived deep acting would be mitigated under a high positive affective state for these members. Model 4 indicated that the three-way interaction effect was statistically meaningful (*b*=0.47, *p*<0.05), and the two-dimensional graph in Figure 3 was also in line with the prediction.

Hypothesis 4 predicted that the negative effect of perceived surface acting on work engagement would be mitigated when positive affect is low for members of low-quality LMX. The three-way interaction term was significant (model 4 of Table 3; *b*=0.35, *p*<0.01). Based on Figure 3, when positive affect was low, the negative effect of perceived surface acting on work engagement was relatively weak since members were already not engaged in their work as we predicted. However, we found that the negative effect was more apparent when the positive affect was high. As a result, hypotheses 3 and 4 were supported.

## Discussion

The purpose of this research is to present how subordinates respond to leaders’ affective display and to advance a framework which demonstrates that, because of perception, personal, and contextual factors, leader’s positive expression can induce members’ work-related motivation. We empirically illustrate the role of positive affect and quality of LMX in strengthening or weakening the effects of perceived deep and surface acting on work engagement. The results confirm the two three-way interaction effects; for members with high-quality LMX, the positive impact of perceived deep acting on work engagement is more strongly demonstrated in a highly positive affective state, while the negative influence of perceived deep acting is weakened when positive affect is high. Conversely, for members with low-quality LMX, the positive effect of perceived deep acting is weakened when positive affect is high, and the negative impact of perceived deep acting decreases when positive affect is low. Based on these outcomes, we found that the quality of LMX serves as a relational context in which affective display occurs between the leader and the members, and that members’ positive affect changes their reactions to the displayed affect.

### Theoretical Implications

Our research makes significant theoretical contributions to the literature with regard to a leader’s affective display, work engagement, and EASI theory. First, we vividly explain the impact of the affective display from the perspective of members and emphasize the importance of considering contextual factors. Since the importance of a leader’s affective display has been highlighted recently (Van Knippenberg and Van Kleef, 2016; Ashkanasy et al., 2017), this research emphasizes members’ perspective and meaningfully reveals its influence. Several studies have focused on the contagion effects, but failed to explain why leaders’ positive affective display do not always effectively induce members’ anticipated work motivation and behavior (Visser et al., 2013; Wang and Seibert, 2015). However, we explained that the members are not simply reacting, but rather complexly responding under the influence of various factors as well as the perception of the displayed affect. Our results show that the characteristics of the expression, the affective state of the member, and the nature of the relationship drive variances in members’ reaction. This suggests that to clearly determine the effects on members, it is imperative to understand how members’ reactions are formed based on their state and relationship with leaders, rather than simply considering valences (e.g., positive or negative).

Second, this study is meaningful in that it comprehensively indicates how members’ work engagement is formed based on their leader from a multilevel perspective. We demonstrated that members successfully experience work engagement from daily interactions with leaders at the within-person level. According to the results, members with high-quality LMX were more likely to experience work engagement from perceived deep acting when their positive affect was high, while members with low-quality LMX were more likely to experience it when positive affect was low. Meanwhile, the experiencing engagement was hampered by perceived surface acting; especially, those with high-quality LMX were more interrupted when positive affect was low, and members with low-quality LMX were more disturbed when positive affect was high. While this is in line with existing studies that emphasized the importance of a leader’s role in work engagement (Vogelgesang et al., 2013; Breevaart et al., 2016), it is an empirical demonstration of how work engagement is shaped from daily affective interactions with the leader. In addition, some studies assumed that positive relationships built at the between-person level will cause positive daily work experience for work engagement (Christian et al., 2011; Breevaart et al., 2015). Our study explains that high-quality LMX works as a relational context, providing members with the opportunities to experience and maintain work engagement. Therefore, we link the field of work engagement to the leadership field by explaining how members’ engagement can be induced by their leader inclusively, considering the between-person-level factor and phenomena at the within-person level.

The third theoretical contribution of our study is that it expands the EASI theory by considering both the within-person-level and between-person-level moderators. The EASI theory and relevant empirical studies underlined the characteristics of the observer in understanding the effects of the affective display, and addressed personality (between level) as the moderator (Van Kleef et al., 2010a; Hideg and Van Kleef, 2017). Furthermore, from these studies, we demonstrate that the responses of the members vary according to their positive affect, and that the affective state of the observer is also a crucial characteristic that should be considered (within level). This is in line with existing research flows suggesting that the effects of affective display should be understood at the within-person level (Gabriel and Diefendorff, 2015; Diefendorff et al., 2019), and the affective state as one of the observer’s characteristics fluctuates every moment like the affective expression, and it acts as a background in the formation of the observer’s response. Therefore, our findings determine that the within-level characteristics of the observer like positive affect, which fluctuate every moment like the affective display, influence the interpersonal effect of affective display significantly. In addition, the study clarifies that quality of LMX is an important context in the affective interactions between leaders and members. Since leaders and members have a lasting relationship, the nature of the relationship affects the effectiveness of the affective display (Gooty et al., 2010). This is similar to the EASI theory, which emphasized the consideration of contextual characteristics in order to understand the social influence of expressed emotion (Van Kleef, 2016). We applied the EASI theory to the leader-member context, arguing that the member’s reaction toward the displayed affect can be varied according to high-quality and low-quality LMX, and they were verified empirically.

Moreover, by conducting three-way interaction effect which is a valuable way to advance the current understanding of how affective expression influences others, we further extend the EASI theory, which argued that the characteristics of displayed affect, observer, and context should be considered in the formation of a member’s response, and focused on how each of them affects individually (Van Kleef, 2016). In particular, researchers tend to focus on one of the various factors but have not launched extensive efforts to address them simultaneously. However, since members’ responses are formed under the influence of multiple factors, this study has validated the three-way interaction effects to capture this phenomenon. Specifically, we have explained why the leader’s positive expression may not always engender members’ motivation, by considering members’ perception of expression, affective state, and established relationships concurrently. This methodological approach provides the lens with which to identify the paradoxical effects of positive expression.

### Practical Implications

Our research provides the following practical implications. The results of this study show that while the leader’s positive affective display plays an important role in members’ work engagement, the mechanism is not simple. The effects were maximized or even appeared more negatively as members’ perceptions, positive affect, and quality of LMX serve as conditions. These results advise organizations to invest in training programs that can enhance the emotional intelligence of leaders in order to ensure effective emotional management of their members. It is not desirable to encourage leaders to simply display positive affect because this expression is an act that consumes leaders’ job resources (Gardner et al., 2009). Meanwhile, developing a leader’s ability to take into account the affective state of members and displaying skills so that they can be perceived as deep acting will effectively manage members’ work motivation. Hence, fostering a leader’s emotional intelligence and interpersonal skills will manage the daily work experience of the members and make a desirable contribution toward improving organizational effectiveness.

Moreover, this study recommends that it is necessary to establish a high-quality LMX for both leaders and members. Our results demonstrate that building a high-quality LMX augments the positive effect of perceived deep acting and buffers the negative effect of perceived deep acting on work engagement. This suggests that establishing high-quality LMX offers leaders the advantage of efficiently managing their members, and also serves as an opportunity to induce positive workplace experiences for members. Accordingly, organizations should pay attention to the relationship between leaders and members. They should support the formation of high-quality LMX, particularly for leaders of newly created teams, and share this information to encourage them to form a high-quality relationship with members.

### Limitations and Directions for Future Research

Our research has several limitations, and based on them, we propose directions for future research. First, since the main variables used in our study were measured by members, doubts could be raised about the common method variances (Podsakoff et al., 2003). To compensate for this potential problem, we performed group-mean centering for all within-personal-level variables and controlled the effect of means. Therefore, our findings are not explained by individual differences and can be seen as the result of the intended within-person-level phenomenon (Sonnentag et al., 2012; Antonakis et al., 2019). Additionally, we made an effort to reduce potential biases by including variables measured by the leader, such as a leader’s extraversion and positive affect in examining the hypotheses (Podsakoff et al., 2003). Nevertheless, to further address these problems, we propose that the future research can use the outcome variables of members measured by the leader, such as member’s voice and OCB.

Although this study logically explored the relationship between variables based on theoretical backgrounds and prior studies, since the measure of within-person-level variables was conducted concurrently, the reverse causality issue can be raised. We acknowledge this limitation and propose that future research should measure variables at different time points to firmly establish the relationship. Specifically, by measuring perceived deep/surface acting and measuring work engagement a few hours later than that, it is possible to clarify antecedents’ role more meaningfully.

This research focused on dealing with direct interactions between leaders and members in real-environment settings. However, there have been many changes in today’s business environment, and many forms of interaction and communication have occurred online. Online affective display differs considerably from actual affective display in direct face-to-face situations (Van Kleef, 2016); the way it is expressed, the way it is conveyed, and the process that affects the others. Therefore, in order to understand the effects of affective display more broadly and reflect today’s environment, it is worthwhile to address the online affective display in the future research.

The purpose of this study is to examine how leaders’ positive affective display influences the work engagement of members, but leaders’ affective display is not limited to that of positive affect. In the workplace, the leader also displays negative affect toward the members, and the effects will also depend on the characteristics of expression, the member’s affective state, and relationship. Therefore, we recommend that the effects of the leader’s negative affective display on members could be addressed together, which can be understood as a different mechanism from that of positive affective display (Liu et al., 2017). Based on this, we hope to enhance the overall understanding of the role of affective display in the leadership process and the influence of affective display on a member’s workplace experiences.

## Conclusion

Given that a leader’s affective display is prevalent in the workplace, it is imperative to understand how it affects members’ motivation and behavior. Our study revealed that the effects on members’ work engagement can be altered depending on whether the expression is perceived as deep acting or surface acting, how quality of LMX was formed, and the extent of positive affect. It appears that the members cognitively judge displayed affect and express reactions based on these characteristics. Our results indicate that the effect of the leader’s positive affective display on a member is complex; therefore, its effect on work engagement is determined by various characteristics.

## Data Availability Statement

The datasets presented in this article are not readily available because it contains personal information, and it is to protect the privacy of participants. Requests to access the datasets should be directed to SH, gener0977@gmail.com.

## Ethics Statement

Ethical review and approval was not required for the study on human participants in accordance with the local legislation and institutional requirements. Written informed consent for participation was not required for this study in accordance with the national legislation and the institutional requirements.

## Author Contributions

SH and MK conceived and designed the research, and drafted and edited the manuscript. SH collected, analyzed, and interpreted the data. MK administered the project. All authors contributed to the article and approved the submitted version.

## Conflict of Interest

The authors declare that the research was conducted in the absence of any commercial or financial relationships that could be construed as a potential conflict of interest.

## Publisher’s Note

All claims expressed in this article are solely those of the authors and do not necessarily represent those of their affiliated organizations, or those of the publisher, the editors and the reviewers. Any product that may be evaluated in this article, or claim that may be made by its manufacturer, is not guaranteed or endorsed by the publisher.
